# Variants in the IL17 pathway genes are associated with atopic asthma and atopy makers in a South American population

**DOI:** 10.1186/s13223-019-0340-7

**Published:** 2019-04-29

**Authors:** Milca de J. Silva, Maria B. R. de Santana, Bruna R. Tosta, Roberta P. Espinheira, Neuza Maria Alcantara-Neves, Maurício L. Barreto, Camila Alexandrina Figueiredo, Ryan dos S. Costa

**Affiliations:** 1 Departamento de Biorregulação, Laboratório de Imunofarmacologia e Biologia Molecular, Instituto de Ciências da Saúde, Universidade Federal da Bahia, Salvador, Bahia Brazil; 2 Departamento de Ciências da Biointeração, Instituto de Ciências da Saúde, Universidade Federal da Bahia, Salvador, Bahia Brazil; 3 Fundação Oswaldo Cruz, Salvador, Bahia Brazil

**Keywords:** Asthma, Atopy, Variants, IL-17 genes

## Abstract

**Background:**

Asthma is a complex disorder with multiple phenotypes which can influence its severity and response to treatment. The T_H_17 lymphocytes producing IL-17A and IL17-F cytokines, may have a role on asthma inflammation. The aim of our study was to evaluate the association between genetic variants in *IL17* pathway genes with asthma and atopy markers.

**Materials and methods:**

Genotyping was performed using a commercial panel in 1245 participants of SCAALA cohort. The study included 91 SNVs in IL-17 pathway genes. Logistic regressions for asthma and atopy markers were performed using PLINK 1.9. In silico analyses were performed using rSNPbase, RegulomeDB, and Gtex portal for in silico gene expression.

**Results and discussion:**

The T allele of rs1974226 in *IL17A* was positively associated with asthma (OR: 1.37; 95% CI 1.02–1.82). Also, the T allele of rs279548 was positively associated with asthma (OR: 1.30; 95% CI 1.02–1.64), atopy (OR: 1.62; 95% CI 1.05–2.50) and increased expression of the *IL17RC* in lung and whole blood tissues. The others genetic variants in the *IL*17 pathways genes were associated with both protection and risk for asthma development as well as with IgE levels.

**Conclusion:**

The genetic variants in IL-17-related genes are associated with the atopic asthma phenotype and IgE production.

## Introduction

About 334 million people worldwide suffer from asthma, and this number tends to increase [2]. It is expected that in 2025, 100 million new asthma cases will occur worldwide [4]; thus, it represents a global public health problem, especially in developing countries where the westernized life style led to an increase of such disease [9]. Asthma is one of the most common chronic inflammatory diseases of lower airways, affecting children and young adults [37]. It has a heterogeneous etiopathogeny which arises from various factors associated with a complex genetic basis as well as several environmental factors and individual variability. All these factors together are responsible for the wide variety of inflammatory phenotypes [10, 19, 27], leading to different response to treatment, making it difficult to establish a specific therapy for each phenotypes since most of them are still unknown [31].

**Fig. 1:**
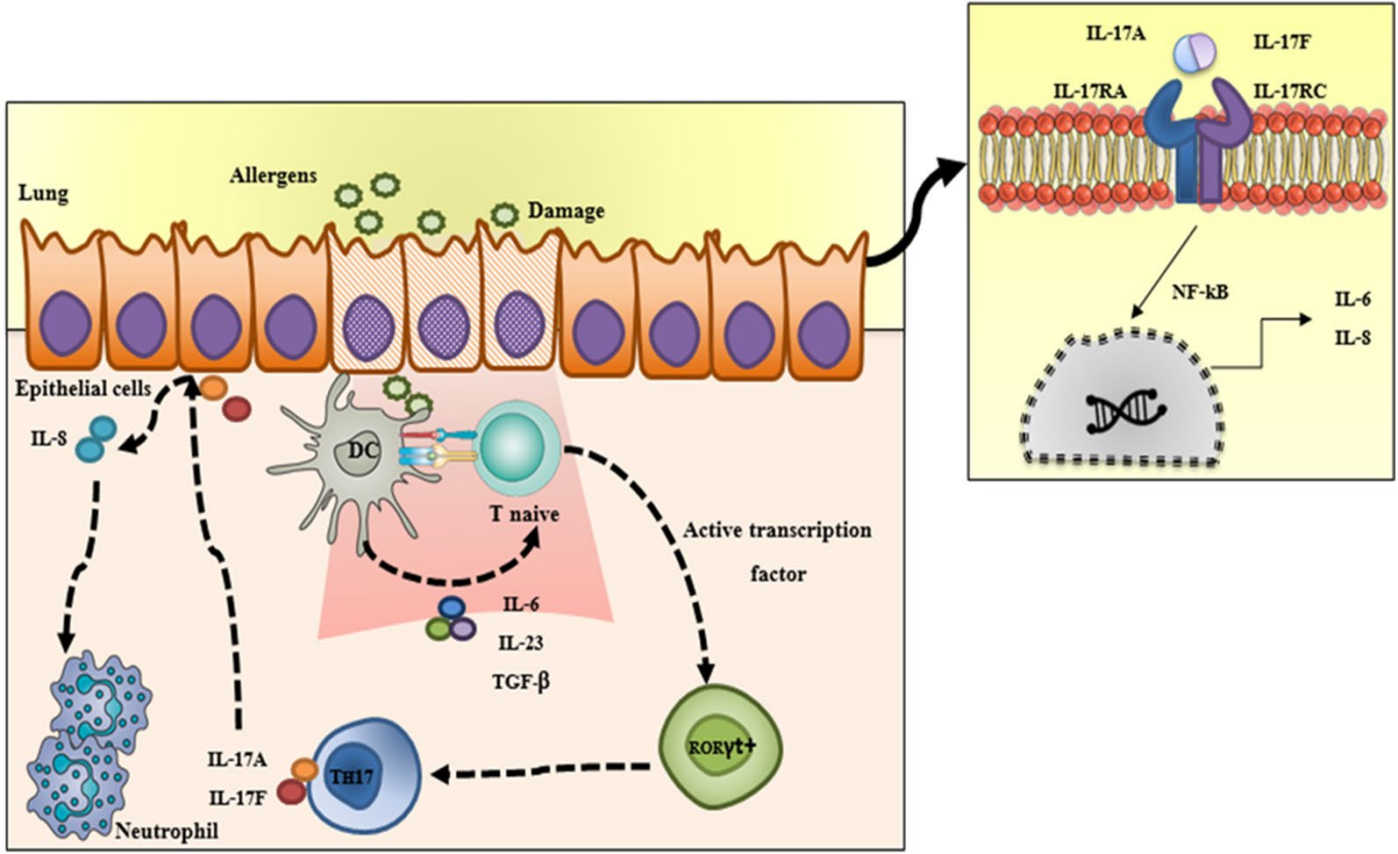
The role of Th17 in non-atopic asthma. In response to allergens and pollutants, epithelial cells produce cytokines that stimulate dendritic cells (DC) priming T CD4+ cells to a pro-inflammatory phenotype by the activation of RORyt transcription factor leading to the differentiation of naïve T CD4+ cells into Th17 effector cells. These cells secrete IL-17A and IL-17F, which act as heterodimers in the IL-17RA and IL-17RC receptors in the epithelial cells surface. Thus, this interaction actives the NFkB that start nuclear transcription of cytokines (IL-6 and IL-8). Also, IL-8/CXCL8 induces neutrophil chemotaxis

Several genes have been associated with distinct asthma phenotypes. Classically, the allergic asthma is orchestrated by T2-type immune response which is related to the development of allergic inflammation inducing IgE release, mucus secretion and eosinophils chemotaxis to the lungs. More recent studies have showed that, part of asthma patients have basal levels of T2-type cytokines in association to no allergic sensitization, known as non-allergic asthma (non-atopic). The non-atopic asthma may be associated with T_H_17 T CD4^+^ lymphocytes [32]. In the presence of allergens and other environmental factors, bronchial epithelial cells are stimulated to synthesize cytokines, among them, IL-6 and IL-23. Such cytokines lead the naïve T cells to activate the transcription factors RORγt and STAT3. Thus, these T cells differentiate into T_H_17 lymphocytes, which in turn will stimulate the release of IL-17-family cytokines, especially IL-17A and IL-17F [22]. These cytokines stimulate the inflammatory response in bronchial epithelial cells through interaction with IL-17RA receptors that act preferably as dimers with the IL-17RC. The IL17RA/IL17RC activation can initiate a signaling cascade leading to production of downstream inflammatory mediators and cytokines (IL-6 and IL8) induced by NF-kB transcription factor [24] (Fig. 1). Recent studies show that IL-17A and IL-17F cytokines are overexpressed in the lung tissue and into the bloodstream of some asthmatics patients [1]. Other evidence also have pointed out that, in asthmatic patients, IL-17A expression is increased in lung tissue, sputum and bronchoalveolar lavage fluid (BALF) [29].

It is well established that genetic polymorphisms play a significant role in asthma and, therefore, interest in this field has grown over the past two decades [5]. In studies involving the Th17 pathway, SNVs in the *IL17A* and the *IL17F* were associated with allergic rhinitis and asthma [36]. The most studied variants in the *IL17F* gene (rs763780 and rs2397084) were associated with asthma [20]. Also, the SNVs rs2275913 and rs2397084 were associated with asthma in children in Tunisia [25] and with asthma and rhinitis in a Portuguese population [34]. In a Asian population, the rs2275913 was associated with the development of asthma after bronchiolitis in infancy [16]. One study also identified variants in *IL17RA* associated with the risk of developing aspirin-intolerant asthma [30]. There are no previous studies associating genetic variants in the *IL17RC*, *RORC* and *NFKB* with asthma, however, it is plausible to believe that variants that may interfere in the regulation of these genes play an important role in asthma.

Collectively, these findings indicate that variations on IL-17 pathway genes may be involved in the pathophysiology of atopic asthma and suggest that the production of IL-17 and its accessory downstream molecules are candidates for asthma susceptibility. Therefore, the aim to this study was to evaluate how variants in IL-17-related genes can influence the development of atopy and asthma in a Latin urban population from Brazil.

## Methods

### Study population

The studied population comprised 1245 children born between 1994 and 2001, originally recruited for Social Changes Asthma and Allergy in Latin America (SCAALA) program in the city of Salvador, BA, Brazil, as previously described [3]. Briefly, demographic and environmental data were collected from standardized questionnaires. Asthma diagnosis was done by the application of a Portuguese translated, ISAAC phase II questionnaire. These questionnaires were applied to each child parents or legal guardian in 2005. Blood samples for laboratory tests and isolation of genetic material were collected and skin prick test were done with several allergens in a field ambulatory. This work has been approved by the National Research Ethics Committee (reference number: 120.616) and free informed consent was properly obtained from the parents or legal guardian of each child.

### Skin prick test and allergens specific IgE (sIgE)

All individuals were submitted to skin puncture tests to common inhalant allergens in our region (mites—*D. pteronyssinus*, *D. farinae*, *B. tropicalis*, cockroach—*P. americana* and *B. germanica*; fungi mix (ALK-ABELLO, São Paulo, Brazil). After 15 min, the wheal diameter was measured. The test was considered positive if the mean of the largest perpendicular diameters (excluding pseudopodia) was at least 3 mm greater than the negative control (Table 1).

**Table 1:** Demographic characteristics and allergy markers of the studied children, according to asthma status

	Total population	Non-asthmatic	Asthmatic	p-value
		N (%) 930 (77.30)	N (%) 273 (22.70)	
Sex	1203			
Male	655	501 (53.9)	154 (56.4)	0.233
Female	548	429 (46.1)	119 (43.6)	0.162
Age group (years)	1203			
≤ 5	438	342 (36.8)	96 (21.5)	0.545
6–7	419	316 (40)	103 (24.1)	0.466
≥ 8	346	272 (76.6)	74 (20.8)	0.419
SIgE seropositivity (≥ 0.70 kU/L)	1246			
At least one allergen	458	325 (34.9)	133 (48.7)	< 0.0001*
*D. pteronyssinus*	270	179 (19.2)	91 (33.3)	< 0.0001*
*B. tropicalis*	416	288 (40)	128 (46.9)	< 0.0001*
*B. germanica*	164	117 (12.6)	47 (17.2)	< 0.0001*
*P. americana*	112	81 (8.7)	31 (11.4)	< 0.0001*
Skin prick test positivity (≥ 3 mm)	1.103			
For at least one allergen	372	272 (29.2)	100 (36.6)	< 0.0001*
*D. pteronyssinus*	193	131 (14.1)	62 (22.7)	< 0.0001*
*B. tropicalis*	268	192 (20.6)	76 (27.8)	< 0.0001*
*B. germanica*	91	70 (7.5)	31 (11.3)	< 0.0001*
Helminthic infections	1.119	184 (19.8)	90 (33)	< 0.0001*

* Chi^2^ test p-value ≤ 0.0001

Determination of specific IgE serum concentrations was performed for *Dermatophagoides pteronyssinus*, *Blomia tropicalis*, *Blattella germanica*, and *Periplaneta Americana* using the ImmunoCAP assay (Phadia Diagnostics AB, Uppsala Sweden). Children with ≥ 0.70 kU/L or greater of specific IgE for any allergen tested were considered to have a positive result [12].

### Asthma symptoms and atopy definitions

Atopy was defined if the child had specific IgE ≥ 0.70 kU/L and/or Skin prick test ≥ 3 mm for at least one tested aeroallergens [12].

Asthma (current wheezing) was defined by having wheezing in the last 12 months, plus at least 1 of the following: (1) diagnosis of asthma ever; (2) wheezing with exercise in the last 12 months; (3) 4 or more episodes of wheezing in the last 12 months; and (4) waking up at night because of wheezing in the last 12 months. All children with asthma and atopy were classified as atopic asthmatics. The others with asthma and without atopy were considered as non-atopic asthmatics.

### Genomic DNA extraction

DNA was extracted from peripheral blood samples according to the protocol Flexigene DNA Kit (Qiagen, Hilden, Germany). All samples were standardized at a gDNA concentration of 5 ng/μL and stored at − 30 °C until use.

### Genotyping

The genotyping was performed using a commercial panel of Illumina BeadChip Human Omni2.5-8 Kit (www.illumina.com) with 2284818 SNVs, through the Consortium EPIGEN-Brazil. The information on the genes position was obtained from National Center for Biotechnology Information (NCBI) (www.ncbi.nlm.nih.gov) and used to localize genetic data corresponding to each gene as following: *IL17A* from NC_000006.11 (52051185.0.52055436) position at the chromosome 6, *IL17F* from NC_000006.11 (52101484.0.52109298) position at the chromosome 6, *IL17RA* from NC_000022.10 (17565849.0.17596584) position at the chromosome 12, *IL17RC* from NC_000003.11 (9958758.0.9975305) chromosome 3, *NFKB* from NC_000004.11 (103422486.0.103538459) chromosome 4, and the *RORC* from NC_000001.10 (151778547.0.151804348) position at the chromosome 1. A total of 79 SNVs were analyzed in this study related to genes on IL-17 pathway. Also, 277 informative markers of ancestry were genotyped and used to correct the population stratification. The selection of these markers was based on the allelic frequency difference above 50% between populations present in the HGDP-CEPH projects and 1000 Genomes.

### Statistical analysis

Were included in this study, markers with genotype call rates > 98%, Hardy–Weinberg equilibrium (HWE) with p > 0.05 and population stratification and frequency of minor allele (MAF) with p > 0.05.

Logistic regressions were applied to estimate odds ratio (OR), permutational-p value, and 95% confidential interval (CI) for the association between SNVs, asthma and atopy markers (skin tests and specific IgE production), adjusted for sex, age, helminth infections and ancestry markers. The analyses were performed using the additive genetic model. A permutational p-value lower than 0.05 was considered statistically significant. The permutation test is performed to test the null hypothesis, a difference in the values is expected under the null hypothesis. Permutation procedures provide a computationally intensive approach to generating significance levels empirically. This test control the false discovery rate in order to solve the problem of multiple comparisons [23], preserving the correlational structure between SNVs [33]. All the analyses above were performed using the software PLINK (version 1.9). The Linkage Disequilibrium among cases and controls was created using Haploview 4.2.

### Genetic risk score for asthma symptoms

The genetic score was performed to determine the risk degree in the presence of more than one allele of certain SNVs associated with asthma symptoms in this study. Therefore, it was included in the analyses only the genotype combination of these variants. The score number was attributed according to the presence of minor allele.

All the genetic scores analyses were performed using the SNPstats web tool (www.snpstats.net/snpstats/). The forest graph was created using the GraphPad Prism 5.0 software.

### In silico functional analyses

To identify the SNVs functions the National Center of Biotechnology Information (NCBI) (www.ncbi.nlm.nih.gov) was accessed, which is an open online platform that contains worldwide data about biotechnology, biomedicine and bioinformatics. It houses databases with specifics material, and among them there is the SNV database (dbSNP). The dbSNP is a helpful tool that provides extensive information about SNVs such as the SNV position, the chromosome, allele frequency for each allele variation; also, whether the SNV is intronic, exonic or near to a gene, whether it is a missense variation, which amino acids is changed. dbSNP was used to gather all the information cited above.

The statistical analyses were made using the method of Benjamini and Hochberg [6], and corrected by Bonferroni test. Here, the *Homo sapiens* organism and the option to evaluated a lists of target genes were chosen.

RegulomeDB (regulomedb.org) is a database for interpretation of regulatory variants in the human genome. It includes high-throughput, experimental data sets from Encyclopedia of DNA Elements (ENCODE) and other sources. RegulomeDB identifies putative regulatory potential and functional variants through computational predictions and manual annotations [7]. The greater evidence for a variant to be located in a functional region the lower the score in RegulomeDB is, as follows: *1a to 1f* scores, SNVs are likely to affect the binding and the expression of a gene target; *2a to 2c* scores, SNVs are likely to affect binding; scores *3a and 3b* are less likely to affect binding; *4*, *5* and *6* scores, SNVs may have minimal binding evidence and; *7* score, there are no data about the function of a certain SNV [7].

rSNPBase (http://rsnp.psych.ac.cn/) is a database that provides annotations focused on regulatory SNVs involved in a wide range of regulation types, including proximal, distal and post-transcriptional regulation, to identify their potentially regulated genes [15], in respect of: (i) *proximal regulation* indicates SNVs that are involved in proximal transcriptional regulation. The (ii) *distal regulation*, SNVs should be involved in distal transcriptional regulation. The (iii) *micro RNA regulation* describes SNVs within mature miRNA and the (iv) *RNA binding protein* are SNVs involved in RNA binding protein-mediated post-transcriptional regulation. Through the rSNPbase platform the information about each genetic variants was accessed in UniProt database, and Ensembl genome browser (wgEncodeUwDgf), a directory containing the downloadable files associated with Encyclopedia of DNA Elements (ENCODE) data sources (Uniport, 2014).

The genotype-tissue expression project GTEx (www.gtexportal.org) of the National Institutes of Health Common Fund establishes a resource database in association with tissue bank made with the objective to study the relationship between gene expression and genetic variation, and other molecular phenotypes, in multiple reference human tissues [35]. Expression quantitative trait loci (eQTL) mapping offers a powerful approach to elucidate the genetic component underlying altered gene expression. Genetic variation can also influence gene expression through alterations in splicing, non-coding RNAs, and RNA stability. Gene expression is differentially regulated across tissues, and many human transcripts are expressed in a limited set of cell types or during a limited developmental stage [35]. From this project information was obtained about gene expression according to each SNV.

## Results

### Characteristics of the study population

77.30% of the participants were non-asthmatic individuals, while 22.70% were asthmatics. No significant difference was found for age range and sex between the two groups. On the other hand, there was a significant statistical difference in specific IgE production for at least one allergen, specific IgE for *D. pteronyssinus*, *B. tropicalis*, *B. germanica* and *P. americana* when comparing the non-asthmatics group with asthmatic group. Also, there was a statistical difference in positive skin prick test response for at least one allergen, *D. pteronyssinus*, *B. tropicalis* and *B. germanica* among the two groups (Table 1).

### SNVs included

We found 150 SNVs for the six genes in the IL-17 pathway. Of these, 26 SNV was excluded by Tests of Hardy–Weinberg equilibrium (HWE) (p ≤ 0.001) and 53 SNVs by minor allele frequency (MAF < 0.05). No sample was excluded by low genotyping (mind > 0.1) or missingness (geno > 0.1). After quality control steps, the study included 79SNVs.

### Variants in *IL17* pathway genes can influence asthma

In the *IL17A*, the T allele of rs1974226 was positively associated with asthma (OR: 1.37; 95% CI 1.02–1.82) (Table 2). In the IL17RC, the T allele of rs279548 (OR: 1.30; 95% CI 1.02–1.64), the T allele of rs11917994 (OR: 1.40; 95% CI 1.09–1.79) and the A allele of rs76234423 (OR: 1.39; 95% CI 1.05–1.83), was positively associated with asthma (Table 2). The allele C of rs6769465 was negatively associated with asthma (OR: 0.60; 95% CI 0.34–0.97) (Table 2). In the *NFKB1*, the A allele of rs35680095 (OR: 1.28; 95% CI 1.02–1.61) and the G allele of rs75071695 (OR: 3.25; 95% CI 1.19–8.87) were positively associated with asthma (Table 2). In the *RORC* gene, the allele C of rs4995918 was positively associated with asthma (OR: 1.28; 95% CI 1.04–1.57) (Table 2).

**Table 2:** Significant associations between SNVs in *IL17A*, *IL17RC*, *NFkB* and *RORγ* genes and asthma symptoms by logistic regression analysis adjusted for sex, age, helminth infections and ancestry markers

SNV	Asthma symptoms			p value	Perm
	Risk allele^a^	OR	95% CI		
*IL17A*					
rs1974226	T	1.37	1.02–1.82	0.03	0.02
*IL17RC*					
rs11917994	T	1.40	1.09–1.79	0.01	0.01
rs76234423	A	1.39	1.05–1.83	0.02	0.03
rs279548	T	1.30	1.02–1.64	0.03	0.04
rs6769465	C	0.60	0.34–0.97	0.03	0.04
*NFKB1*					
rs35680095	A	1.28	1.02–1.61	0.03	0.03
rs75071695	G	3.25	1.19–8.87	0.02	0.01
*RORC*					
rs4995918	C	1.28	1.04–1.57	0.01	0.02

*SNV* single nucleotide variants, *OR* odds ratio, *CI* confidence interval, *Perm* permutational-p-value

^a^Alternative/polymorphic allele

### Variants in *IL17* can influence atopic makers

In *IL17RA*, the allele C of rs10483089 was negatively associated with SPT for at least one allergen (OR: 0.76; 95% CI 0.60–0.97 (Table 3). In *IL17RC*, the A allele of rs115461448 was positively associated with anti-*D. pteronyssinus* IgE (OR: 3.09; 95% CI 1.20–8.00) and SPT for one least one allergen (OR: 2.58 and 95% CI 1.01–6.62) (Tables 3 and 4). The allele A of rs77569961 was negatively associated with production of anti-*D. pteronyssinus* IgE (OR: 0.69 and 95% CI 0.51–0.95), sIgE for *B. tropicalis* (OR: 0.74; 95% CI 0.57–0.97) and positive SPT for *D. pteronyssinus* (OR:0.68; 95% CI 0.47–0.98) (Tables 3 and 4). The allele A of rs279545 was positively associated with sIgE production for at least one allergen (OR: 1.35; 95% CI 1.03–1.76), sIgE for *D. pteronyssinus* (OR: 1.26; 95% CI 1.04–1.53) and sIgE for *B. tropicalis* (OR: 1.23 95% CI 1.04–1.50) (Table 4). These variants are not in linkage disequilibrium (Fig. 3d).

**Table 3:** Significant associations between SNVs in *IL17A*, *IL17F*, *IL17RA*, *IL17RC*, *NFkB* and *RORγ* and skin prick tests (SPT) for common aeroallergens by logistic regression analysis adjusted for sex, age, helminth infections and ancestry

SNV	Risk allele^a^	OR	95% CI	p value	Perm
Positive SPT for one of the seven allergens					
*IL17RA*					
rs10483089	C	0.76	0.60–0.97	0.02	0.02
*IL17RC*					
rs115461448	A	2.58	1.01–6.62	0.04	0.04
*NFKB1*					
rs4647992	T	1.50	1.08–2.05	0.01	0.02
rs73837255	G	0.63	0.42–0.93	0.02	0.02
Positive SPT for *P. Americana*					
*IL17RC*					
rs115461448	A	4.36	1.65–11.47	0.002	0.001
Positive SPT for *B. tropicalis*					
*NFKB1*					
rs4647992	T	1.61	1.15–2.26	0.01	0.01
rs909331	A	1.44	1.02–2.04	0.03	0.04
rs73837241	A	0.62	0.40–0.95	0.02	0.03
rs73837255	G	0.61	0.39–0.96	0.03	0.04
*RORC*					
rs11204894	T	1.33	1.06–1.67	0.01	0.01
rs7540799	T	1.54	1.08–2.19	0.01	0.01
rs3790515	T	0.74	0.58–0.96	0.02	0.03
Positive SPT for *B. germanica*					
*IL17RC*					
rs7627880	G	1.34	1.01–1.99	0.04	0.04
*NFKB1*					
rs72929590	A	2.62	1.17–5.86	0.01	0.01
rs72931412	G	2.52	1.10–5.90	0.03	0.01
Positive SPT for *D. pteronyssinus*					
*IL17RC*					
rs77569961	A	0.68	0.47–0.98	0.03	0.03
*NFKB1*					
rs73837255	G	0.33	0.17–0.65	0.001	0.001
rs73837241	A	0.47	0.27–0.81	0.01	0.01
rs4647992	T	1.56	1.06–2.27	0.02	0.02
*RORC*					
rs3790515	T	0.62	0.46–0.85	0.002	0.003
rs11578418	A	1.41	1.05–1.90	0.02	0.02
rs11204894	T	1.30	1.01–1.68	0.04	0.04

*SNV* single nucleotide variants, *OR* odds ratio, *CI* confidence interval, *Perm* permutational p-value

^a^Alternative polymorphic allele

**Table 4:** Significant associations between SNVs in *IL17A*, *IL17F*, *IL17RA*, *IL17RC*, *NFkB* and *RORγ* genes with anti-aeroallergens IgE production by logistic regression analysis adjusted for sex, age, helminth infections and ancestry markers

SNV	Risk allele^a^	OR	95% CI	p value	Perm
Positive for at least one allergen					
*IL17RC*					
rs279545	A	1.35	1.03–1.76	0.02	0.02
rs7627060	G	1.20	1.02–1.42	0.02	0.02
Positive for *D. pteronyssinus*					
*IL17RC*					
rs279545	A	1.26	1.04–1.53	0.01	0.02
rs115461448	A	3.09	1.20–8.00	0.01	0.01
rs77569961	A	0.69	0.51–0.95	0.02	0.02
*NFKB1*					
rs4647992	T	1.64	1.18–2.30	0.004	0.003
rs909331	A	1.51	1.08–2.12	0.01	0.01
rs28491436	T	1.37	1.01–1.86	0.04	0.04
rs73837255	G	0.50	0.31–0.80	0.004	0.005
rs73837241	A	0.57	0.40–0.89	0.01	0.01
*RORC*					
rs1521186	A	1.24	1.01–1.51	0.03	0.03
Positive for *B. tropicalis*					
*IL17RC*					
rs279545	A	1.23	1.04–1.50	0.01	0.03
rs77569961	A	0.74	0.57–0.97	0.02	0.03
*NFKB*					
rs73837255	G	0.64	0.44–0.93	0.02	0.01
rs4647992	T	1.40	1.03–1.91	0.03	0.03
rs73837241	A	0.70	0.49–0.99	0.04	0.03
*RORγ*					
rs11578418	A	1.30	1.02–1.65	0.02	0.02
rs11204894	T	1.27	1.04–1.56	0.01	0.01
Positive for *B. germanica*					
*NFKB1*					
rs4647992	T	1.64	1.10–2.42	0.01	0.01
rs909331	A	1.52	1.02–2.28	0.03	0.04

*SNV* single nucleotide variants, *OR* odds ratio, *CI* confidence interval, *Prem* permutational-p-value

^a^Alternative/polymorphic allele

**Fig. 2:**
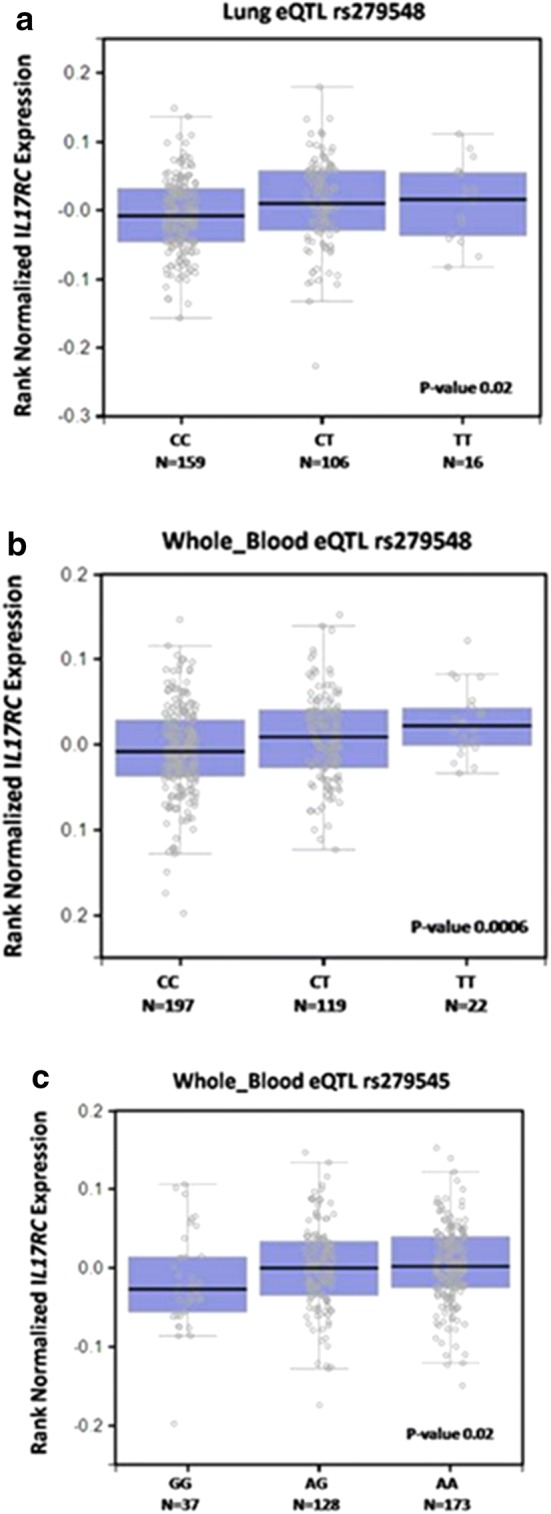
Expression levels of *IL17RC* in lung and whole blood by genotype using the Gtex portal

In *NFKB1*, the allele G of rs73837255 was negatively associated with anti-*D. pteronyssinus* and anti-*B. tropicalis* IgE production, SPT for *D. pteronyssinus* (OR: 0.69; 95% CI 0.51–0.95), for *B. tropicalis* (OR: 0.61; 95% CI 0.39–0.96) and for at least one tested allergen (OR: 0.63; 95% CI 0.42–0.93) (Tables 3 and 4). The A allele of rs73837241 was negatively associated with sIgE production for *D. pteronyssinus* (OR: 0.57; 95% CI 0.40–0.89) and for *B. tropicalis* (OR: 0.70; 95% CI 0.49–0.99) and SPT for *D. pteronyssinus* (OR: 0.62; 95% CI 0.40–0.95) (Tables 3 and 4). These SNVs were not in linkage disequilibrium (Fig. 2f). The allele T of rs4647992 was positively associated with anti-*D. pteronyssinus* (OR: 1.64; 95% CI 1.18–2.30), *B. germanica* (OR: 1.64; 95% CI 1.10–2.42) and *B. tropicalis* IgE production (OR: 1.61; 95% CI 1.15–2.26) (Table 4).

**Fig. 3:**
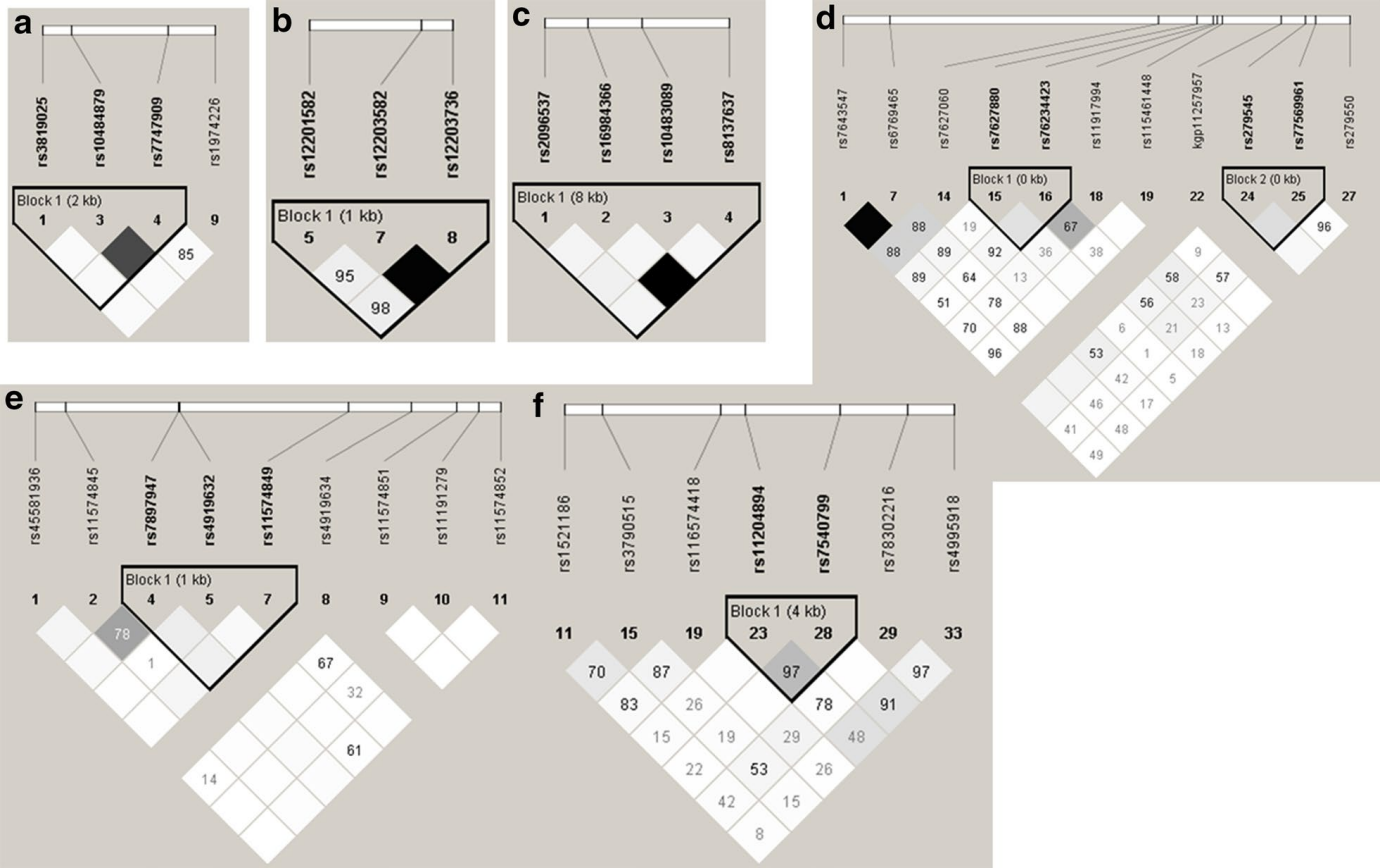
Pairwise linkage disequilibrium (LD) using the D′ statistic test for the *IL17A* gene (**a**) *IL17F* gene (**b**) *IL17RA* gene (**c**) *IL17RC* gene (**d**) *RORC* (**e**) and *NFKB1* (**f**). Intensity of shading indicates the degree of confidence in the D′ value

In *RORC*, the allele A of rs11578418 was positively associated with sIgE production against *B. tropicalis* (OR: 1.30; 95% CI 1.02–1.65) (Table 4), and positively associated with SPT for *D. pteronyssinus* (OR: 1.41; 95% CI 1.05–1.90) (Table 3). The allele T of rs11204894 was positively associated with sIgE anti-*B. tropicalis* (OR: 1.27; 95% CI 1.04–1.56) (Table 4) and positively associated with SPT for *D. pteronyssinus* (OR: 1.30; 95% CI 1.01–1.68) and for *B. tropicalis* (OR: 1.33 and 95% CI 1.06–1.67) (Table 3). Moreover, the rs11578418 and rs11204894 were in total linkage disequilibrium (Fig. 2e). The allele T of rs7540799 was positively associated with SPT for *B. tropicalis* (Table 3). These SNVs (rs11204894 and rs7540799) were in high linkage disequilibrium (Fig. 3e). The allele T of rs3790515 was negatively associated with SPT for *D. pteronyssinus* (OR: 0.62 and 95% CI 0.46–0.85) and for *B. tropicalis* (OR: 0.74; 95% CI 0.58–0.96) (Table 3), this variant was in total linkage disequilibrium with rs78703675 (Fig. 3).

### Variants can increases the risk of asthma symptoms

Initially all genotypes combination of the rs1974226, rs11917994, rs76234423, rs279548, rs35680095, rs75071695 and rs4995918 were analyzed (data not shown). It was found that the risk of asthma susceptibility was increased only when the alleles that acted as risk factors (rs76234423, rs279548, and rs3568009) were presented together.

Genetic score analyses showed that among the individuals, the most frequent genotype was that one which has all wild-type alleles (26.52%) in the rs35680095 (G/G), rs76234423 (G/G) and rs279548 (C/C); these individuals were the reference group (score 0). Individuals were grouped according to the amount of minor allele they presented. The total of subjects included in the score “1” was 529, representing 45.5% of total population. The score “2” were present in 23.92% of population. The score “3” appeared in 5.82% of the subjects. The score “4” composed a minor part of subjects, only 0.72%. And, the least frequent score was the score “5”, with only two individuals (0.16%) (Table 5).

**Table 5:** The genotypes combination and the corresponding gene score of SNVs positively associated with asthma included in the gene risk analyses

Genotypes^a^			N/(%)^b^	Gene score^c^
rs35680095	rs76234423	rs279548		
G/G	G/G	C/C	328 (26.52)	0
A/G	G/G	C/C	209 (16.90)	1
G/G	A/G	C/C	127 (10.30)	
G/G	G/G	T/C	193 (15.60)	
A/G	A/G	C/C	65 (5.25)	2
G/G	A/G	T/C	47 (3.80)	
A/G	G/G	T/C	120 (9.70)	
A/A	G/G	C/C	30 (2.42)	
G/G	A/A	C/C	6 (0.49)	
G/G	G/G	T/T	28 (2.26)	
A/A	A/G	C/C	11 (0.89)	3
A/A	G/G	C/T	20 (1.61)	
G/G	A/G	T/T	4 (0.32)	
A/G	G/G	T/T	13 (1.05)	
A/G	A/G	T/C	25 (2.02)	
A/A	A/G	T/C	3 (0.24)	4
A/A	G/G	T/T	4 (0.32)	
A/G	A/A	T/C	1 (0.08)	
A/A	A/A	C/C	1 (0.08)	
A/A	A/A	T/C	1 (0.08)	5
A/A	A/G	T/T	1 (0.08)	

^a^Genotypes combination of each SNVs analyzed, the sequence of alleles represents the A1/A2

^b^Number of individuals in population with the genotype, and the correspondent frequency

^c^Attributed score for each genotype

**Fig. 4:**
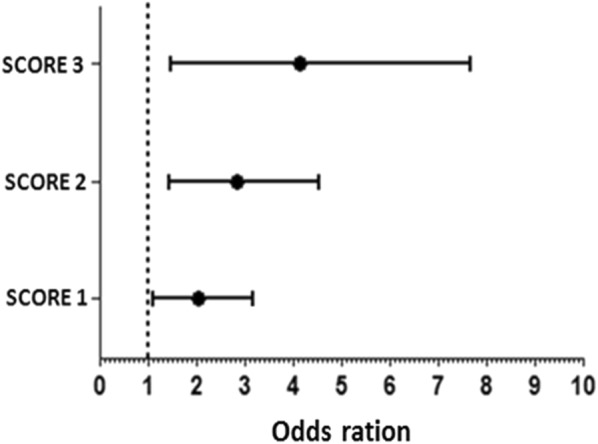
The degree of genetic polymorphisms (gene scores) was positively associated with asthma symptoms. The dotted line represents the reference value. The black circles are corresponding to OR values and the horizontal lines represent the scale of confidence interval. The p-value and the exact values of OR and CI are bellow the lines. All data are statistical significant

Statistical analyses between the gene score and asthma symptoms showed that, in the presence of one polymorphic allele (score 1), the risk of asthma symptoms was increased (OR: 1.86; 95% CI 1.09–3.15) when adding another risk allele the OR did not change too much (OR: 95% CI 1.44–4.52). However, in the presence of three minor allele (score 3), the risk of symptoms of asthma is approximately three times larger (OR: 3.25; 95% CI 1.38–7.65). All analyses were compared with the reference group (Fig. 4). Because of the low numbers of individuals with the score 4 and score 5 in our population, the gene score analysis could not be performed.

### In silico *IL17RC* expression is increased in the presence of variants

The eQTL was examined in silico to obtain expression of the each gene according to the genotype in lung and whole blood when available in Gtex portal. All SNVs described in this study were evaluated. Nevertheless, no difference in gene expression was found for *IL17A*, *IL17F*, *IL17RA*, *NFKB1* and *RORC* according to genotype for the SNVs explored in this study (data not showed). On the other hand, gene expression analysis for *IL17RC* (Fig. 2), the T allele of rs279548 increased the *IL17RC* expression in lung and whole blood (p-value ≤ 0.05); the A allele of rs279545 also increases the *IL17RC* expression in the whole blood (Fig. 2a–c), (p-value ≤ 0.05).

### Regulatory features of SNVs

Table 6 summarizes the results of rSNPBase in which regulatory features of retrieved SNVs in four (i–iv) regulation manners are summarized as ‘Yes’ or ‘No’. All SNVs in the *IL17A*, *IL17F*, *IL17RA* and *IL17RC* interfere in proximal transcription regulation, which is related to regulatory elements associated with DNA accessibility. In *NFKB* and *RORC* the variants involved in this process were few. Most of SNVs involved in the distal regulation were present in *IL17RC*, *NFKB and RORC*; this SNVs can modify the chromatin interactions. All SNVs in *NFKB* are involved with miRNA regulation, only SNVs in this gene were involved with this type of regulatory feature. The miRNA are important to control the levels of RNA expression. Also, most of the SNVs operate in the post-transcriptional RNA binding protein mediated regulation.

**Table 6:** In silico functional analyses using rSNPBase and RegulomeDB platforms according to significant SNVs (in association analysis) for *IL17A*, *IL17F*, *IL17RA*, *IL17RC*, *NFKB1* and *RORC* genes

SNV	Proximal regulation	Distal regulation	miRNA regulation	RNA binding protein mediated regulation	RegulomeDB score^a^
*IL17A*					
rs1974226	Yes	No	No	Yes	4
Il17F					
rs12203582	Yes	No	No	Yes	5
rs12203736	Yes	No	No	Yes	7
Il17RA					
rs2096537	Yes	No	No	No	7
rs76234423	Yes	Yes	No	Yes	4
rs6769465	Yes	Yes	No	Yes	5
rs10483089	Yes	No	No	No	5
IL17RC					
rs11917994	Yes	Yes	No	Yes	4
rs76234423	Yes	Yes	No	Yes	4
rs279548	Yes	Yes	No	Yes	7
rs6769465	Yes	Yes	No	Yes	7
rs279545	Yes	Yes	No	Yes	1b
rs115461448	Yes	Yes	No	Yes	5
rs7627880	Yes	Yes	No	Yes	6
rs77569961	Yes	Yes	No	Yes	2b
NFKB1					
rs73837255	No	No	Yes	No	3b
rs73837241	No	Yes	Yes	No	5
rs4647992	Yes	Yes	Yes	No	5
rs909331	No	Yes	Yes	No	3a
rs28491436	No	No	Yes	No	5
RORC					
rs1521186	No	Yes	No	Yes	5
rs11578418	No	Yes	No	Yes	2b
rs7540799	No	Yes	No	Yes	2b
rs11204894	No	Yes	No	Yes	4
rs3790515	No	No	No	Yes	5
rs4995918	Yes	Yes	No	Yes	4
rs78703675	Yes	No	No	Yes	2b

*SNV* single nucleotide variants

^a^RegulomeDB score annotation

For the significant variants found in this study, RegulomeDB was used to identify putative regulatory potential and functional SNVs (Table 6). This database has a score ranging from 1 to 7, as previously described in Methods section (Table 3). Few variants were found with high involvement in expression mechanisms (score 1–3); most of the variants were involved in transcription biding factors.

All the SNVs studied herein had some level of evidence in terms of having an impacting gene expression.

## Discussion

In our study, it was shown for the first time that variants in IL-17 pathway can interfere on asthma and atopy in a Brazilian population.

Studies have demonstrated the important role of IL-17 pathway in pulmonary diseases [22], specially in asthma, considering that the overexpression was demonstrated of IL-17A and IL17F in lungs of non-atopic asthmatic patients [1]. Also, Molet et al. [28] demonstrated that in asthmatic subject, IL-17A is increased in lungs, sputum and bronchoalveolar fluid. In our work it was found that the variants in genes from IL-17 pathway can increase the risk for developing asthma and atopy; this fact is probably due to an increase of IL-17 levels gene expression in lung of asthmatics subjects. All variants that increase the risk of developing asthma are involved in proximal regulation, and this type of regulation is largely associated with gene expression [15].

The actions of IL-17A and IL-17F depend on the appropriate link to their receptors IL17RA and IL17RC [13]. It was showed that variants in *IL17RC* are associated with increased risk of asthma (rs11917994, rs76234423 and rs279548) and atopy (rs279545, rs7627060, rs115461448, rs77569961) development. Among variants in *IL17RC*, the T allele of rs279548 increases the expression of *IL17RC* in lung and whole blood and the A allele of rs279545 increases the expression of *IL17RC* in whole blood. Function analyses suggest that they can be involved in proximal, distal, transcriptional regulation and post-transcriptional regulation. Moreover, this variant opens the chromatin in chr6:52055240-52055390 position, on T_H_17 cells lineage. Also, the post transcriptional regulation of the SNV is involved in the regulation of *ELAVL1* (ELAV like RNA binding protein 1); the protein encoded by this gene is closely related with the mRNAs stabilization and gene expression [26].

The pro-inflammatory signalization of *IL17RA* and *IL17RC* are largely linked to NF-kB [8]. NF-κB is a transcription factor expressed in numerous cell types, which plays a key role in the pro-inflammatory cytokines production [11]. Variants were found in *NFKB1* (rs35680095 and rs75071695) elevating the risk of asthma and atopy development; furthermore, according to our results, all variants in this gene are involved in inactivation of miRNA. Interestingly, genetic score analyses demonstrated that combination variants in *IL17RC* and *NFKB1* together increased the risk of asthma in comparison with the presence of these variants alone. It was found that variants in *NFKB1* have an important role in asthma according to studies that indicate enhanced NF-κB pathway activation in asthmatic tissues [14], and the NF-kB expression is increased in the airway epithelium of asthmatic humans [21].

Another transcription factor related with asthma is the RORγt; it has been described as a major factor that determines the phenotype of airway inflammation and steroid sensitivity in asthma [17]. In our study a variant (rs4995918) was also found in this gene increasing the asthma risk.

In other way, some variants were also found as a protect factor against asthma (rs6769465) and atopy (rs77569961, rs73837255, rs73837241 and rs73837255). These SNVs are located in non-coding region and variants in these regions can alter the RNA splicing process and interfere with exons junctions impacting directly on the protein translation [18]. Moreover, it was found that some of these protecting SNVs block the distal transcriptional, which can lead to a low expression of pro-inflammatory genes involved in asthma and atopy.

In conclusion, variant in genes of IL-17 pathways may influence in the development course of asthma pathology and atopy. Also, more studies are necessary to further elucidate the potential role of *IL17* pathways genes on asthma and atopy, and how it could be a strategy to control this disease.

## Abbreviations

BAL: bronchoalveolar lavage
CI: confidence interval
eQTL: expression quantitative trait *loci*
gDNA: genomic deoxyribonucleic acid
IgE: immunoglobulin E
IL-17: interleukin 17
LD: linkage disequilibrium
MAF: minor allele frequency
HWE: Hardy–Weinberg equilibrium
NFkB: nuclear kappa B factor
OR: odds ratio
RORγ: related orphan receptor gamma
SNV: single nucleotide variant
